# A new species of the genus ***Gaeolaelaps*** (Acari, Mesostigmata, Laelapidae) from Iran

**DOI:** 10.3897/zookeys.277.4741

**Published:** 2013-03-15

**Authors:** Mohammadreza Kavianpour, Alireza Nemati, Dariusz J. Gwiazdowicz, Farhan Kocheili

**Affiliations:** 1 Department of Plant Protection, Faculty of Agriculture, Shahid Chamran University of Ahvaz, Iran; 2 Department of Plant Protection, Faculty of Agriculture, University of Shahrekord, Iran; 3 Poznan University of Life Sciences, Department of Forest Protection, Wojska Polskiego 71C, 60–625 Poznań, Poland

**Keywords:** Taxonomy, Acari, Mesostigmata, Laelapidae, *Gaeolaelaps*

## Abstract

The Genus *Gaeolaelaps* Evans & Till, 1966 is currently one of the largest genera of the family Laelapidae Berlese. The known representatives of this genus are active predators of small invertebrates such as other mites, insect eggs and nematodes. *Gaeolaelaps iranicus* Kavianpour & Nemati **sp. n.**, was collected from soil and litter in various parts of Iran. The description and figures of this species are given. A key to the *Gaeolaelaps* species of Iran is provided.

## Introduction

The mite family Laelapidae Berlese includes hundreds of species that are free-living predators in soil, as well as many others that have varying degrees of association with other animals, both vertebrates and invertebrates (Faraji and Halliday 2009). The genus *Gaeolaelaps* Evans & Till is currently one of the largest genera of the family Laelapidae. Some species of this genus, such as *Gaeolaelaps aculeifer* Canestrini, *Gaeolaelaps oreithyiae* Walter and Oliver, and *Gaeolaelaps gillespiei* Beaulieu, are aggressive predators of nematodes and immature arthropods (Beaulieu 2009, Smiley et al. 2003, Trach 2012, Walter and Moser 2010).

*Gaeolaelaps* was considered at different taxonomic levels by authors: as a species group (Van Aswegen and Loots 1970); as a subgenus of *Hypoaspis* sens. lat. (Karg 1962, 1982, 1989, 1987, Faraji et al. 2008), and as a distinct genus (Lapina 1976, Ryke 1963, Hyatt 1964, Rosario 1981, Walter and Oliver 1989). We herein consider *Gaeolaelaps* as a genus. So far, a total of twelve species were reported from Iran: *Gaeolaelaps aculeifer* (Canestrini), *Gaeolaelaps nolli* (Karg), *Gaeolaelaps kargi* (Costa), *Gaeolaelaps queenslandicus* (Womersley), *Gaeolaelaps angusta* (Karg), *Gaeolaelaps angustiscutatus* (Willmann), *Gaeolaelaps praesternalis* (Willmann), *Gaeolaelaps minor* (Costa), *Gaeolaelaps postreticulatus* (Xu & Liang), *Gaeolaelaps deinos* (Zeman), *Gaeolaelaps oreithyiae* (Walter & Oliver), and *Gaeolaelaps glabrosimilis* (Hirschmann, Bernhard, Greim & Götz) (Mosaddegh 1997, Nemati et al. 2000, Kamali et al. 2001, Hadad Irani-Nejad et al. 2003, Mosavi et al. 2004, Noei 2007, Faraji et al. 2008, Babaeian et al. 2010, Nemati and Babaeian 2010, Ramezani and Nemati 2010). During our survey of soil and litter habitats in Iran we discovered a thirteenth species, new to science, which we describe in this paper.

## Materials and methods

Mites were collected from various soil and litter samples from different parts of Esfahan, Chaharmahal Va Bakhtiari and Khuzestan provinces in Iran. Mites were extracted from samples using Berlese funnels, placed in lactic acid at 55 ^°^C for clearing and then mounted in Hoyer’s medium on permanent microslides. All specimens were examined under a phase contrast microscope. Line drawings were made by use of a drawing tube and figures were performed with Corel X-draw software, based on the scanned line drawings. Seven specimens were used for most characters measurements. All the measurements are given in micrometers (μm). The dorsal setae notation followed that of Lindquist and Evans (1965). The term lyrifissures and pore are used to refer to slit-shaped and circular or oval-shaped cuticular openings, respectively. We have attempted to identify all pore-like structures, but we acknowledge that some may have been overlooked. The holotype and some of the paratypes are deposited in the Acarological Laboratory, Department of Plant Protection, Agricultural College, Shahrekord University Shahrekord, Iran. Some paratypes are deposited in the Senckenberg Museum fur Naturkunde Gorlitz Am Museum 1 02826 Gorlitz Germany and Acarological Laboratory, Department of Plant Protection, Agricultural College, Shahid-Chamran University, Ahwaz, Iran.

## Results

Genus *Gaeolaelaps* Evans & Till, 1966

**Type species:** *Laelaps aculeifer* Canestrini (1884), by original designation (Evans and Till 1966).

### Gaeolaelaps iranicus

Kavianpour & Nemati sp. n.

urn:lsid:zoobank.org:act:2E62A4A8-00F3-4F54-BA82-E10A85DDBCF3

http://species-id.net/wiki/Gaeolaelaps_iranicus

**Figures 1–2.**
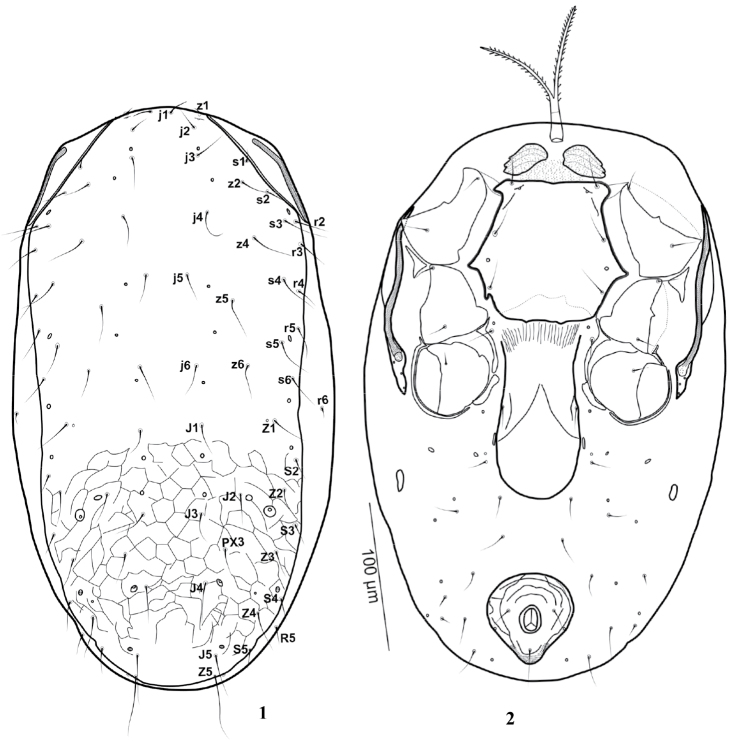
*Gaeolaelaps iranicus* Kavianpour & Nemati sp. n., Female. **1** Dorsum **2** Venter.

Figures 1 [Fig F2] –8

#### Specimens examined.

Holotype, female, soil, Shahreza (32°03'N, 51°54'E, alt. 1777 m), Esfahan Province, Iran, 11 July 2010; coll., M. Kavianpour.

Paratypes: Females, soil from different parts of Shahreza, Esfahan province, and from different parts of Chaharmahal Va Bakhtiari and Khuzestan provinces, Iran, with the following data: Esfahan province, Shahreza: eight females (32°07'N, 51°55'E, alt. 1725 m), 22 August 2010; one female (32°06'N, 51°54'E, alt. 1747 m), 22 August 2010; three females (32°02'N, 51°53'E, alt. 1795 m), 1 September 2010; one female (32°01'N, 51°53'E, alt. 1799 m), 5 September 2010; seven females (32°01'N, 51°53'E, alt. 1800 m), 20 March 2011; five females (32°01'N, 51°53'E, alt. 1806 m), 4 April 2011; three females (32°02'N, 51°51'E, alt. 1827 m), 11 June 2011; three females (31°39'N, 51°55'E, alt. 2220 m), 9 July 2011; three females (32°00'20"N, 51°52'54"E, alt. 1823 m), 17 July 2011; one female (31°56'N, 51°44'E, alt. 1963 m), 4 August 2011.

Chaharmahal Va Bakhtiari province, Shahrekord (32°19'N, 50°51'E, alt. 2206 m),three females, soil and litter, 2012, coll., A. Nemati.

Khuzestan province, Baghmalek (31°31'N, 49° 53'E, alt. 707 m), two females, soil; Ghaletol (31°37'N, 49°53'E, alt. 885 m), two females, soil; Izeh (31°49'N, 49°52'E, alt. 845 m), two females, soil, 2012, coll., A. Nemati.

#### Diagnosis.

Female; with small size (330–400); dorsal shield with 36 pairs of setae (*PX2* and *S1* missing), *PX3* setae present between *J* and *Z* series, only two pairs of marginal setae (*r6*, *R5*) in soft lateral cuticle, considerably shorter than dorsal shield setae; peritremes long, extending to anterior of setae *s1*; *leg IV*: tarsus (91-99: basitarsus + telotarsus), (*al1-3*, *av1-2*, *pl1-3* and *pv1-2* thickened, *ad2-3* and *pd2-3* slender and very elongate: *ad2-3* (0.55–0.57 and 0.66-0.69 × the length of tarsus IV respectively) and *pd2-3* (0.74–0.83 and 0.94-1.02 × the length of tarsus IV respectively).

#### Description of the female

(n = 7). Figures 1–8.

***Dorsal idiosoma***. Fig 1. Dorsal shield oval-shaped, 330-400 long, width at level of setae *r3* 170-195; reticulation more distinct posterior to setae *j6*; shield with 36 pairs of thin and simple setae, 21 pairs on podonotal region (*j1-6*; *z1*, *z2*, *z4-6*; *s1-6*; *r2-5*; *z3* missing) and 15 pairs on opisthonotal region (*J1-5*, *Z1-5*, *S2-5*), including *PX3* between *J* and *Z* series; *PX2* and *S1* missing. Dorsal setae vary in length, with opisthonotal setae generally slightly longer than podonotal setae: *j1* (14-20), *j2* (16-21), *j3* (20-33), *j4* (21-34), *j5* (20-28), *j6* (22-34); *z1* (8-14), *z2*, *z4*, *z5*, *z6* (22-34); *s1* (13-16), *s2* (15-26), *s3* (26-38), *s4*-*6* (27-36); *r2*, *r3*, *r4* and *r5* (21-31); *J1,J2* (21-28), *J3* (18-28), *J4* (25-34), *J5* (29-39); *Z1* (25-34), *Z2*-*Z3* (18-30), *Z4* (26-39), *Z5* longest (40-50), *S2*-*S4* (14-20), *S5* (18-25). Cuticle between dorsal and ventral side of body bearing *r6* (between *s6* and *Z1*) and *R5* (between *S4*-*S5*), length 8 and 12 µm long, respectively. Podonotal and opisthonotal regions with 9 and 10 pairs of lyrifissures and pore-like structures, respectively, as shown in Fig. 1.

***Ventral idiosoma*** (Fig 2). Tritosternum with tubular base (23-26) and pilose laciniae (70-75). Pre-sternal area granulated, with a pair of distinct, although poorly sclerotized plates. Sternal shield with smooth surface, 111-114 long, 114-118 wide (at level of projection between coxae II-III), with very small notch anteromedially; posterior margin irregular, almost straight. Sternal setae smooth, *st1*, *st2* and *st3* (26-29), *iv1* slit-like, located slightly behind *st1*; *iv2* pore-like, between *st2*-*st3*. Setae *st4* (23-26) and pore-like *iv3* located on integument. Tongue-shaped genital shield 127-135 long (including hyaline flap at base of posterior margin of sternal shield), 57-60 wide, bearing 1 pair of setae (*st5* = 21-28) and a pattern of inverted v-shaped lines; paragenital pores (*iv5*) on soft integument near genital setae. Anal shield pyriform, reticulated, 65-70 long, 62-67 wide, post-anal seta (34-42) longer than paranal setae (18-22). Cribrum like a strip of teeth, extending laterally to level of post-anal seta. Opisthogastric surface with: 1 pair of suboval metapodal plates (12-15 × 3-6); 2 pairs of minute platelets (between metapodal plate and paragenital platelet); 1 pair of narrow, elongate paragenital platelets; 9 pairs of smooth setae, *ZV1*-*4* and *JV1*-*5* 16-29 long; and 7 pairs of pore-like structures, plus 1 pair on lateral margin of anal shield.

Stigma surrounded by short, narrow, pointed stigmatal plate, which extends posteriorly past level of mid-coxae IV (a distance ca. thrice diameter of stigma). Peritremes long, extending to anterior of setae *s1*. Narrow endopodal platelet present mesad coxae III–IV. Narrow exopodal plate surrounding coxae IV, and small exopodal plate between coxae II–III.

**Figures 3–6. F2:**
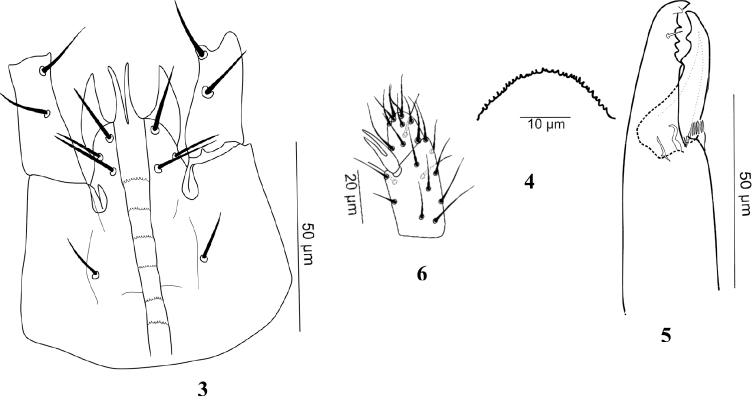
*Gaeolaelaps iranicus* Kavianpour & Nemati sp. n., Female. **3** Hypostome **4** Epistome **5** Chelicera **6** Apotele.

***Gnathosoma***. Hypostome (Fig. 3) with 3 pairs of similar smooth simple setae; *h1*, *h3* (20-26), *h2* (15-17). Palpcoxal setae 15-18 long. Deutosternal groove with 6 rows of 8-10 denticles; corniculi normal, horn-like. Epistome rounded with fine denticulations at anterior margin (Fig. 4). Chelicerae (Fig. 5) normal for genus, arthrodial processes developed, moveable digit (40-45) with 2 teeth, middle article (105-120), fixed digit with 4 teeth + offset tooth (gabelzahn), setaceous pilus dentilis small. Palp chaetotaxy normal (sensu Evans and Till 1966), with simple setae except *al* on femur thickened, spine-like, *al1* and *al2* of genu thickened, *al1* with tip rounded and *al2* spine-like; palp apotele two-tined (Fig. 6).

***Legs***. Tarsi I–IV with claws and ambulacra. Legs I (432-442) and IV (382-397), longer than legs II (283-291) and III (255-270). Chaetotaxy of all leg segments normal for *Gaeolaelaps* (sensu Beaulieu 2009). Chaetotaxy of legs II and IV as shown in igures 7 and 8, respectively. Setae on legs I and III simple, slender; some setae on legs II and IV thickened or elongate, as follows. Leg II: femur (*al2* and *av2* short, slightly thickened, *pd1* elongate, slender), genu (setae *av1* and *pv1* slightly thickened), tibia (*av1* and *pv1* slightly thickened), tarsus (all setae thickened, except *al2-3*, *pl2-3*, *ad3* and *pd3*). Leg IV: trochanter (seta *pv2* thickened), femur (seta *pd* thickened, *ad1* elongate, slightly thickened), tibia (setae *al1*, *av1* and *pv1* thickened), tarsus (*al1-3*, *av1-2*, *pl1-3* and *pv1-2* thickened, *ad2-3* and *pd2-3* very elongate).

***Insemination structures***. Not seen.

#### Male.

Unknown.

#### Etymology.

The name of this new species refers to the currently known geographic range of the mite.

#### Notes.

*Gaeolaelaps iranicus* sp. n. is differentiated by the following combination of characters: small size (330–400 long), the presence of four very long setae on tarsus IV (*ad2*, *ad3*, *pd2* and *pd3*), and 36 pairs of dorsal setae, with the absence of *PX2* and *S1* on the opisthonotal part of the dorsal shield, and the presence of two pairs of *r*-setae (*r6*, *R5*) on soft cuticle, which are considerably shorter than the dorsal shield setae.

The dorsal shield chaetotaxy is not always properly described, especially in older descriptions. However, species with as few as 36 pairs of setae appear to be rare, but some species do lack one pair of *PX* setae, as found in *Gaeolaelaps iranicus*. Some specimens of *Gaeolaelaps fishtowni* (Ruf and Koehler 1993) appear to lack seta *PX3* but this species is larger than *Gaeolaelaps iranicus* (565-653), has thickened spine or spur-like setae on the femur, genu, tibia and tarsus of leg II and tarsus IV, and the dorsal shield is posteriorly attenuated.

*Gaeolaelaps vanpletzeni* (Van Aswegen and Loots 1970) has 38 pairs of setae on dorsal shield, lacking the *R* series, and has only one pair of *PX* setae (*PX2)*, while *Gaeolaelaps iranicus* sp. n. has 36 pairs of dorsal setae, with *R5* and *PX3* present. *Gaeolaelaps spiniseta* (Barilo 1991) has 38 pairs of dorsal setae, with one pair of *PX* setae (*PX2*), a larger size (505–525), and with elongate spine-like setae on tarsus IV.

*Gaeolaelaps kargi* (Costa) has two elongate setae on tarsus IV, *S1* and *PX2-3* present, the postanal seta as long as the para-anal setae, and lacks elongate setae on genu IV. In contrast, *Gaeolaelaps iranicus* sp. n. has four elongate setae on tarsus IV, lacks *S1* and *PX2* , the post-anal seta is considerably longer than the para-anal setae, and has two elongate setae (*ad1* and *pd1*) on genu IV.

*Gaeolaelaps nolli* (Karg) has two elongate setae on tarsus IV, a short peritreme (extending to the middle part of coxae II) and has *z3*, *PX2* and *S2*, while *Gaeolaelaps iranicus* sp. n. has four elongate setae on tarsus IV, a longer peritreme and lacks z3, *PX2* and *S2*.

Some characters of *Gaeolaelaps iranicus* sp. n. seem to be unique, but again poor descriptions hinder comparisons from the literature. For example, the presence of elongate setae on the femur, genu and tibia of leg IV (*ad1* on femur, *ad1* and *pd1* on genu, *ad2-3* and *pd2-3* on tarsus) is likely, in combination, to be unique in *Gaeolaelaps*. The length of leg segments and the relative lengths of the setae and their form may also be of taxonomic value. Hence we also report the following data relating to these characters: femur IV (80-82), *ad1* (0.77–0.87× the length of femur); genu IV (55-57), *ad1* (0.77-0.91 × the length of genu), *pd1* (0.77-0.86 × the length of genu); tarsus IV (91-99: basitarsus + telotarsus), (*al1-3*, *av1-2*, *pl1-3* and *pv1-2* thickened, *ad2-3* and *pd2-3* slender and very elongate: *ad2-3* (0.55-0.57 and 0.66-0.69 × the length of tarsus IV respectively) and *pd2-3* (0.74-0.83 and 0.94-1.02 × the length of tarsus IV respectively).

**Figures 7–8.**
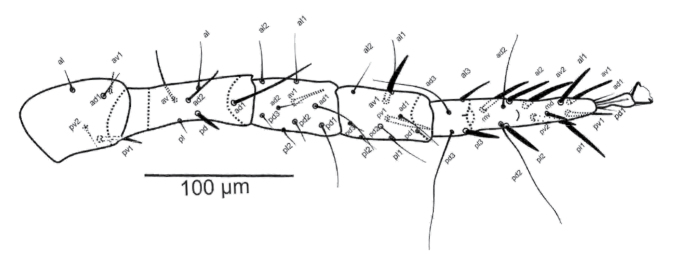
*Gaeolaelaps iranicus* Kavianpour & Nemati sp. n., Female. **7** Leg II **8** Leg IV.

### Key to the *Gaeolaelaps* of Iran (females)

**Table:** 

1	Peritreme short, reaches to middle level of coxa II	*Gaeolaelaps nolli* (Karg, 1962)= *Gaeolaelaps praesternalis* sensu Evans & Till, 1966*
–	Peritreme longer, reaches at least to anterior level of coxa I	2
2	Dorsal shield attenuated, with sudden constriction caudally	3
–	Dorsal shield normal or less attenuated posteriorly	5
3	Leg II with thickened spines, especially a thick spine on femur II; moveable digit of chelicerae with 2 teeth; palp tarsal claw 3-tined	4
–	Without thickened spines on leg II and femur II; movable digit of chelicerae with 2 larger and several smaller teeth; palp tarsal claw 2-tined	*Gaeolaelaps angustiscutatus* (Willmann, 1951)
4	Leg I shorter than idiosoma; epistome with a row of equal denticles; dorsal shield without a curvature	*Gaeolaelaps angusta* (Karg, 1965)
–	Leg I longer than idiosoma; epistome with 2 teeth longer than the others; dorsal shield with a curvature in posterior part	*Gaeolaelaps queenslandicus* (Womersley, 1956)
5	Tarsus of leg IV with spine-like setae thicker than normal	6
–	Tarsus of leg IV without spine-like setae	8
6	Some podonotal setae elongate, twice the length of opisthonotal setae, fixed digit of chelicerae with 12-14 teeth	*Gaeolaelaps aculeifer* (Canestrini, 1883)
–	Podonotal setae not elongate, *Z5* approximately equal to *J5*; fixed digit of chelicerae with less than 12 teeth	7
7	iv2 slit-like; *al1* on femur IV short and spine-like	*Gaeolaelaps deinos* (Zeman, 1982)
–	iv2 pore-like; *al1* on femur IV elongate and simple	*Gaeolaelaps oreithyiae* (Walter & Oliver, 1989)
8	Seta *st1* located in presternal region off sternal shield	*Gaeolaelaps minor* (Costa, 1968)
–	Seta *st1* located on sternal shield	9
9	Genital shield with diagonal parallel lines that meet together in the median area of the shield; sternal shield with concave posterior margin; with 3 unpaired setae between *J* series; seta *z3* present	*Gaeolaelaps glabrosimilis* (Hirschmann, Bernhard, Greim & Gotz, 1969)
–	Genital shield without diagonal parallel lines; sternal shield with relatively straight posterior margin; without unpaired setae between *J* series; without seta *z3*	10
10	Dorsal shield setae short, none of them reach to base of next setae; without elongate setae on tarsus IV	*Gaeolaelaps praesternalis* (Willmann, 1949) after Karg (1971)**
–	Dorsal shield setae relatively long, some of them reach to the base of next setae; with elongate setae on tarsus IV	11
11	With setae *PX2-3* and *S1*; with 2 elongate setae on tarsus IV, para-anal setae slightly shorter than or as long as post-anal seta	*Gaeolaelaps kargi* (Costa, 1968)
–	Without setae *PX2* and *S1*; with 4 elongate setae on tarsus IV, post-anal seta considerably longer than para-anal setae	*Gaeolaelaps iranicus* sp. n.

* We suspect *Gaeolaelaps postreticulatus* (Xu & Liang) recorded by Montazeri et al. (2011) from Iran is a junior synonym of *Gaeolaelaps nolli*.

** The second author has examined the following materials: Two females, soil, Izeh, Khuzestan province, Iran, 2010.

## Supplementary Material

XML Treatment for Gaeolaelaps iranicus
